# Dr. Simpson on Actæa Racemosa

**Published:** 1861-07

**Authors:** 


### DR. SIMPSON ON ACTÆA RACEMOSA.

*From the London Medical Times.*

The *Actœa*, or *Cimicifuga Racemosa*, has been long spoken of as a remedy for rheumatism, and particularly in the more acute forms of the disease. In the edition of Gray's Supplement to the Pharmacopœas, published in 1821, you will find the use of it in rheumatism stated. Latterly it has been employed by some American physicians as their most valuable remedy in acute rheumatic fever. My very intelligent and excellent friend, Dr. Voris, of Rochelle, New York, told me two years ago, that since employing the tincture of actæa in rheumatic fever—and it is a very common disease in his district—he had seen the disease almost always cut short before the eighth or tenth day; the drug acting apparently as a simple antidote to the rheumatic poison, and curing without diuresis, diaphoresis, or any other discharge. The American physicians give a strong tincture of the root in acute rheumatism in doses of thirty to sixty drops every two, three, or four hours. It may be given, if you choose, along with alkaline salts, or other anti-rheumatic drugs. I have found it, in my case, repeatedly cure an attack of lumbago with wonderful rapidity. Some of the American practitioners who have written upon actæa, have spoken of its use in terms that are, no doubt, exaggerated. Thus, Dr. Davis, of Chicago, says, that, after much experience, he has no more doubt of the efficacy of actæa in the early stage of acute rheumatism, than he has of the power of vaccination as a preventive of small-pox. But our American brethren have used actæa also extensively in chorea and other anomalous forms of nervous disease. However unlike rheumatism chorea may look to the superficial observer, yet the able investigations of Dr. Begbie and other pathologists, have shown, as you are aware, an analogy, if not an identity, between the blood poison which produces rheumatism and that which produces chorea. Dr. Physic and Dr. Jesse Young, about thirty or more years ago, recommended actæa strongly in chorea. Latterly, Drs. Lindsey, Kirkbride, Otto, and others, have published their experience in favor of the same drug in this disease. In a case of anomalous and severe chorea of long standing, which was under my care some months ago, the actæa was given with excellent effect. The patient had been previously treated, both in France and in this country, with zinc, iron, arsenic, and all the usual remedies applied in this malady. But I have made all this long episode regarding the actæa, not so much to speak of its use in the preceding diseases, as of its use in puerperal hypochondriasis and depression. A lady, the mother of several children, was twice the subject of the most painful mental despondency a month or two after delivery. On one of these occasions she was confined in London, and had the advice of several eminent physicians; but the disease took a very long and tiresome course, seemed to defy entirely all remedies, and gradually and very slowly terminated. On the last occasion on which the attack occurred, this patient was confined under my care here, and went home to England some weeks subsequently, perfectly well. She returned, however, in about a month, to Edinburgh in the lowest possible state of depression, a perfect picture of mental misery and unhappiness. I tried many plans to raise her out of this dark and gloomy state. All failed. At last, fancying from some of her symptoms and complaints that there might be a rheumatic element in the affection, I ordered her fifteen drops of the tincture of actæa thrice a day. After taking one dose she refused to continue it, as the drug had a taste so similar to laudanum, and as all opiates had always made her worse. On being re-assured that there was no opiate in the medicine, she recommenced, without any faith, however, in the results, as she had, in a great measure, lost faith in all remedial means. When I saw her next, some eight or ten days afterwards, she was altered and changed in a marvelous degree, but all for the better. On the third or fourth day, as she informed me, the cloud of misery which had been darkening her existence suddenly began to dissolve and dispel; and in a day or two more she felt perfectly herself again in gaiety, spirits, and energy. But nothing would induce her to give up the actæa for six or eight weeks longer; and the last time she passed through Edinburgh she told me that she had prescribed her own remedy to more than one melancholic subject with nearly as great success as she had used it in her own case.

